# Designing trials of complex interventions for efficacy and mechanisms evaluation

**DOI:** 10.1186/1745-6215-12-S1-A143

**Published:** 2011-12-13

**Authors:** Richard A Emsley, Jonathan Green, Graham Dunn

**Affiliations:** 1 Health Sciences Research Group, School of Community Based Medicine, The University of Manchester, Manchester, UK; 2 Mental Health and Neurodegeneration Research Group, School of Community Based Medicine, The University of Manchester, Manchester, UK

## Objectives

There have been recent developments in statistical methods for assessing the mechanisms through which a complex intervention acts, with particular applications in mental health trials. This literature has focused on causal mediation analysis, which differs from statistical mediation analysis by its explicit acknowledgement and exposition of the necessary assumptions, particularly that of no unmeasured confounding between the putative mediators or mechanisms and outcomes. Instrumental variable estimation procedures can produce valid causal estimates even in the presence of this unmeasured confounding, but the problem then becomes finding valid instruments to identify the statistical models. So far, however, little attention has been given to the issue of designing trials of complex interventions with this analysis in mind.

## Methods

We introduce the instrumental variables estimation procedure, and discuss some possible methods for obtaining instruments. We then propose four alternative designs for trials of complex interventions in mental health which could produce these potential instruments:

1. “Waiting list control”, where the control group receive the intervention after a specified period of time;

2. “Innocuous vaccine”, where all participants are measured on a baseline variable strongly related to the putative mechanism;

3. “Parallel trials”, where a concurrent set of trials are run in parallel with different interventions but common measures;

4. “Mediated moderation”, where a potential instrumental variable is included as a baseline measure.

## Results

We demonstrate how these trial designs can help validate the causal mediation analysis and strengthen some of the fundamental assumptions underpinning it. We give examples with illustration of the “parallel trials” design in a randomised trial of cognitive behaviour therapy in psychosis, and a “mediated moderation” design in parent mediated treatment trial in child psychiatry.

## Conclusions

Well-designed complex intervention trials should not only consider evaluating the efficacy of the intervention but also the putative mechanisms through which it acts. Adopting a suitable design for the trial both enables and strengthens the assessment of this mechanistic analysis.

